# Factors Related to Decline of Renal Function in Patients with Chronic Hypoparathyroidism †

**DOI:** 10.3390/jcm14165732

**Published:** 2025-08-13

**Authors:** Elena López-Mezquita Torres, Antonia García-Martín, María del Carmen Andreo-López, Victoria Contreras-Bolívar, Cristina García-Fontana, Beatriz García-Fontana, Manuel Muñoz-Torres

**Affiliations:** 1 Endocrinology and Nutrition Unit, University Hospital Clínico San Cecilio, 18016 Granada, Spain; elopezmt@correo.ugr.es (E.L.-M.T.); mcandreo21@correo.ugr.es (M.d.C.A.-L.); victoria.contreras.sspa@juntadeandalucia.es (V.C.-B.); cgfontana@fibao.es (C.G.-F.); bgfontana@fibao.es (B.G.-F.); mmt@mamuto.es (M.M.-T.); 2 Institute of Health Carlos III, CIBER of Frailty and Healthy Aging (CIBERFES), 28029 Madrid, Spain; 3 Department of Medicine, University of Granada, 18016 Granada, Spain; 4 Instituto de Investigación Biosanitaria de Granada (ibs.GRANADA), 18012 Granada, Spain

**Keywords:** chronic hypoparathyroidism, chronic kidney disease, cardiovascular disease, parathormone analogues

## Abstract

**Background/Objectives:** Patients with chronic hypoparathyroidism are at increased risk of kidney complications. Also, chronic kidney disease is associated with increased cardiovascular risk. The aim was to analyze the factors that influence kidney function, including cardiovascular diseases (CVD), in a cohort of patients with chronic hypoparathyroidism. **Methods:** This was a retrospective longitudinal study that included 100 patients with chronic hypoparathyroidism. **Results:** The estimated glomerular filtration rate (eGFR) was associated with the duration of disease (*p* = 0.014). During follow-up, a significant decrease in eGFR was observed over time (*p* < 0.001), and changes in the eGFR were associated with the duration of disease (*p* < 0.001). We found that the eGFR was lower in patients with urolithiasis (*p* = 0.003), hypertension (*p* < 0.001), type 2 diabetes (*p* = 0.031) and dyslipidemia (*p* < 0.001). In total, 14% of patients had a chronic kidney disease (CKD), and these patients had a longer duration of disease (*p* < 0.001). The percentage of patients with urolithiasis (*p* = 0.003), nephrocalcinosis (*p* = 0.008), hypertension (*p* = 0.005), type 2 diabetes (*p* < 0.001), dyslipidemia (*p* < 0.001), coronary heart disease (*p* = 0.008), and arrhythmia (*p* < 0.001) was higher in patients with CKD. Logistic regression models showed that disease duration was associated with CKD (OR = 1.11; 95% CI [1.03–1.22]; *p* = 0.008). We used ROC curves to assess the usefulness of disease duration as a marker of CKD, and the AUC was 0.850 (95% CI 0.763–0.937, *p* < 0.001). A duration of disease > 15.5 years had a sensitivity of 85.7% and a specificity of 71.9% for a diagnosis of CKD. **Conclusions:** The duration of disease appears to be a predictor of the presence of renal dysfunction in patients with chronic hypoparathyroidism. In addition, the coexistence of CVD factors could result in greater renal damage.

## 1. Introduction

Hypoparathyroidism is a rare disease characterized by hypocalcemia and relatively high phosphate levels due to low parathyroid hormone (PTH) levels. Chronic hypoparathyroidism is defined as a disease duration of more than 12 months. The most common cause is accidental damage to the parathyroid glands following cervical surgery, most often in relation to thyroidectomy. Less common causes include genetic mutations and autoimmune diseases [1,2].

Chronic hypoparathyroidism treated with standard therapy using oral calcium and active vitamin D is associated with an increased risk of renal deterioration [3]. Compared to individuals without chronic hypoparathyroidism, patients with it are also at increased risk for marked declines in renal function, chronic kidney disease (CKD) stage progression, and development of end-stage renal disease [4,5]. Long-term therapy can cause hypercalcemia, hypercalciuria, and increased calcium phosphorus product, which might increase the risk of urolithiasis, nephrocalcinosis, and CKD [5,6,7]. Treatments that replicate the normal effects of PTH while reducing the need for conventional therapy could help prevent damage to renal function in patients with chronic hypoparathyroidism [8].

CKD was associated with a high risk of coronary heart disease, heart failure, arrhythmias, and sudden cardiac death. Also, arterial stiffness is increased in CKD [9]. Thus, the main cause of mortality in patients with kidney failure is cardiovascular disease. In addition, classical risk factors such as hypertension, type 2 diabetes, and dyslipidemia are implicated in the early stages of CKD [10].

PTH appears to have both direct and indirect effects on the cardiovascular system. Indirect effects could occur through changes in plasma levels of calcium, and PTH can exert a direct effect through the PTH receptor 1 [11], which is expressed by smooth vascular and endothelial cells, cardiomyocytes, and the cardiac conduction system [12]. PTH has been shown to exert a positive chronotropic and inotropic effect and the ability to act as a vasodilator [13]. Additionally, calcium itself is highly important for cardiac contraction [14]. In exploratory analysis, treatment with PTH analogues was associated with a lower risk of incident cardiovascular disease compared to conventional treatment in patients with hypoparathyroidism [15].

In this context, we aimed to analyze the factors that influence kidney function, including CVD, in a cohort of patients with chronic hypoparathyroidism.

## 2. Patients and Methods

### 2.1. Study Population

This retrospective longitudinal study included 100 patients with chronic hypoparathyroidism older than 18 years, followed between 2019 and 2024, in the Endocrinology and Nutrition Unit of the University Hospital Clinic San Cecilio of Granada (Spain). The time of follow-up was the time between diagnosis and the last visit. A diagnosis of hypoparathyroidism required albumin-adjusted serum calcium levels below the reference limits (8.8–10.4 mg/dL), along with undetectable or inappropriately low PTH levels on at least two determinations, separated by a minimum of two weeks. Permanent hypoparathyroidism was considered if the condition persisted for more than 12 months. Patients with pseudohypoparathyroidism were excluded. Prior to collecting data from the medical history, informed consent was obtained from each patient, ensuring their voluntary involvement. This study received ethical approval from the Granada Provincial Research Ethics Committee in February 2019 to collect previous data from the patients’ medical history, and it adhered to the principles outlined in the World Medical Association Declaration of Helsinki.

### 2.2. Clinical Evaluation

Demographic data, etiology, duration of hypoparathyroidism (period between diagnosis and the last visit), and clinical complications associated with hypoparathyroidism, such as urolithiasis, nephrocalcinosis, hypercalciuria, or CKD, were collected. To consider the diagnosis of urolithiasis and/or nephrocalcinosis, imaging tests such as renal/abdominal ultrasound, abdominal computed tomography (CT), or abdominal X-ray were required to confirm them. In total, 54% of patients had renal imaging techniques performed. In the analysis, only cases that had imaging techniques for the diagnosis of urolithiasis and/or nephrocalcinosis were analyzed.

Data regarding chronic diseases associated with cardiovascular risk, such as arterial hypertension, type 2 diabetes, and dyslipidemia, and cardiovascular disease (CVD), such as coronary heart disease, cerebrovascular disease, peripheral arterial disease, and arrhythmia (especially atrial fibrillation) were collected. Data on patient treatment regimens were also collected and analyzed: dose of calcium supplements (calcium carbonate mg/day), active vitamin D (calcitriol μg/day), cholecalciferol and calcifediol supplements (UI/day), magnesium supplements (mg/day), and thiazides (mg/day).

### 2.3. Biochemical Test

Among the biochemical parameters, the baseline and most recent values of creatinine (mg/dL) and estimated glomerular filtration rate (eGFR) (mL/min/1.73 m^2^) were collected. Changes in the eGFR were evaluated based on absolute value (ΔeGFR = eGFR at last visit—eGFR at diagnosis) and corrected for the duration of follow-up (ΔeGFR/yr = ΔeGFR/time in years). Also, the most recent values found in the clinical history for serum calcium (mg/dL), serum albumin (g/dL), albumin-adjusted serum calcium levels (mg/dL) (total calcium mg/dL+ (4—serum albumin g/dL) × 0.8), PTH (pg/mL), serum phosphorus (mg/dL), serum magnesium (mg/dL), serum 25 OH vitamin D (ng/mL), and 24 h calciuria (mg/24 h) were collected and were measured using standard automated laboratory techniques. Hypercalciuria was defined as a 24-h urine calcium level > 250 mg/24 h in females and 300 mg/24 h in males at any point during follow-up. CKD is defined as an estimated glomerular filtration rate (eGFR) below 60 mL/min/1.73 m^2^. The eGFR was calculated using the Chronic Kidney Disease Epidemiology Collaboration equation (CKD-EPI) according to the equation published by Inker et al. in 2021 [16].

### 2.4. Statistical Analysis

Analyses were performed using SPSS version 28.0.1.0 software (IBM Corp., Armonk, New York, NY, USA) and RStudio. A Kolmogorov–Smirnov test was used to test the normality of the distribution of the continuous variables. The data for continuous variables were expressed as means (standard deviation, SD), and categorical variables were presented as percentages. Associations between continuous variables were described by Pearson’s and Spearman’s correlation coefficients. The mean values between groups were compared using the unpaired Student’s *t*-test and the U Mann–Whitney test for continuous distributed variables. The χ^2^ test and Fisher’s exact test were used to compare categorical variables between groups. A multiple linear regression model was performed to determine the variables independently associated with the eGFR (dependent variable). To identify duration of hypoparathyroidism as an independent predictor of CKD, defined as eGFR < 60 mL/min/1.73 m^2^, logistic regression models were used. The duration of chronic hypoparathyroidism as an estimator of CKD was evaluated using the receiver operating characteristic (ROC) curve. The Youden index was calculated to assess the effectiveness of the prognostic marker (duration of chronic hypoparathyroidism), allowing the selection of a threshold value or optimal cut-off point for the biomarker of interest. Statistical significance was set at *p* < 0.05 (two-tailed).

## 3. Results

### 3.1. Characteristics of Population of Study

Data were available on a total of 100 patients. The average age of patients was 57 years (range 29–86 years), and 85% were females. Most patients had acquired hypoparathyroidism following surgery (89%). Almost all our patients were being treated with calcium supplements (97%) and activated vitamin D (92%). Table 1 shows the characteristics of the study population.

### 3.2. Estimated Glomerular Filtration Rate (eGFR)

The eGFR was correlated with age (r = −0.591, *p* < 0.001), duration of disease (r = −0.390, *p* < 0.001), albumin-adjusted serum calcium levels (r = −0.335, *p* < 0.001), and serum phosphorus (r = 0.253, *p* = 0.012) from the last visit.

**Figure 1:**
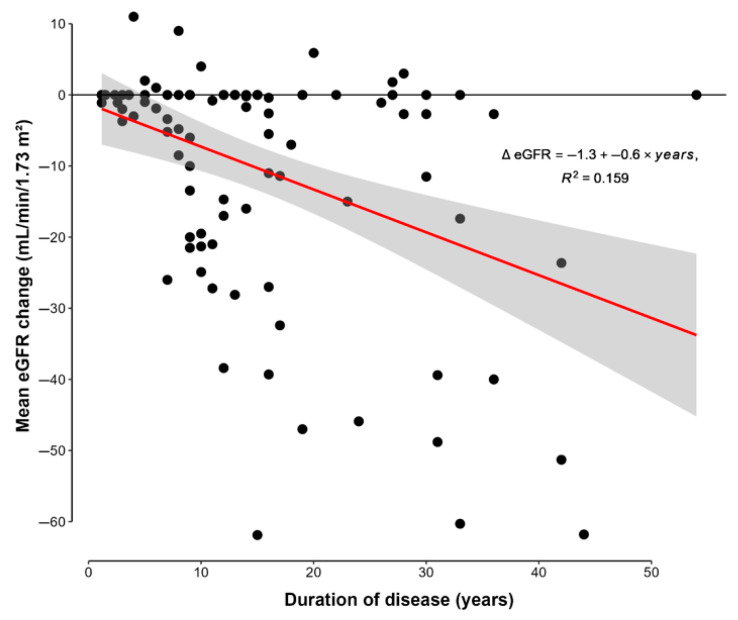
Scatter plots showing the regression analysis with association of mean change in eGFR (ΔeGFR = eGFR at last visit—eGFR at diagnosis) with duration of disease. eGFR: estimated glomerular filtration rate.

During follow-up, a significant decrease in the eGFR was observed between the baseline and the last visit (88.4 ± 5.4 vs. 78 ± 17.5 mL/min/1.73 m^2^, *p* < 0.001). In regression analysis, changes in the eGFR (ΔeGFR = eGFR at last visit—eGFR baseline) were associated with the duration of disease (β = −0.60; 95% CI [−0.89, −0.32]; *p* < 0.001) after adjustment for age, albumin-adjusted serum calcium levels, and serum phosphorus (Figure 1).

We found a diminished eGFR value in patients with urolithiasis (61.4 ± 22.2 vs. 84.6 ± 8.7 mL/min/1.73 m^2^, *p* = 0.005), hypertension (70 ± 20.5 vs. 83.2 ± 12.5 mL/min/1.73 m^2^, *p* < 0.001), type 2 diabetes (65.1 ± 22.3 vs. 81 ± 14.5 mL/min/1.73 m^2^, *p* = 0.004), and dyslipidemia (70 ± 21.3 vs. 84.3 ± 9.6 mL/min/1.73 m^2^, *p* < 0.001). In linear regression analysis, the association with the eGFR remained significant with age (β = −0.426, *p* < 0.001), duration of disease (β = −0.226, *p* = 0.014), and serum creatinine (β = −0.786, *p* < 0.001), independent of albumin-adjusted serum calcium levels and serum phosphorus.

### 3.3. Chronic Kidney Disease (CKD)

At diagnosis, based on the calculated eGFR, 78% of our patients had normal renal function with an eGFR > 90 mL/min/1.73 m^2^, whereas the eGFR was between 60 and 90 mL/min/1.73 m^2^ in 22% and < 60 mL/min/1.73 m^2^ in 0%. However, during follow-up, in the last visit, 40% of our patients had normal renal function with an eGFR > 90 mL/min/1.73 m^2^, whereas the eGFR was between 60 and 90 mL/min/1.73 m^2^ in 46% and < 60 mL/min/1.73 m^2^ in 14%. There was a significant change (*p* < 0.001) in the diagnosis of CKD over time.

The characteristics and differences between patients with eGFR ≥ 60 mL/min/1.73 m^2^ and patients with eGFR < 60 mL/min/1.73 m^2^ are shown in Table 2.

Patients with CKD were older (*p* < 0.001) and had a longer duration of disease (*p* < 0.001), higher albumin-adjusted serum calcium levels (*p* < 0.001), and lower serum phosphorus (*p* = 0.002). ΔeGFR and ΔeGFR/yr were higher (*p* < 0.001) in subjects with CKD. Also, the percentage of patients with urolithiasis (*p* = 0.003), nephrocalcinosis (*p* = 0.008), hypertension (*p* = 0.005), type 2 diabetes (*p* < 0.001), dyslipidemia (*p* < 0.001), coronary disease (*p* = 0.008), and arrhythmia (*p* < 0.001) were higher in subjects with CKD.

### 3.4. Duration of Disease

Logistic regression models were employed to assess the association between the duration of disease with CKD in patients with chronic hypoparathyroidism. The results indicated that the duration of disease was associated with the deterioration of renal function (OR = 1.11; 95% CI [1.03–1.22]; *p* = 0.008) after adjustment for age and the eGFR at diagnosis. The risk of CKD (eGFR < 60 mL/min/1.73 m^2^) increases by 11% for each year of hypoparathyroidism progression.

**Figure 2:**
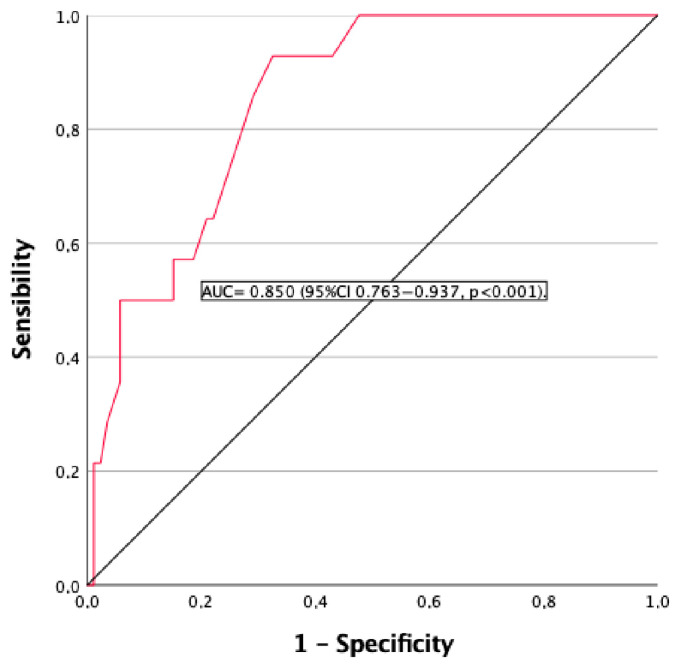
ROC curve for the usefulness of duration of chronic hypoparathyroidism as a marker of eGFR < 60 mL/min. AUC = 0.850 (*p* = 0.001). The AUC indicates the probability of CDK risk, and values greater than 0.70 indicate a good predictive performance. ROC: receiver operating characteristic; AUC: area under the curve.

In the receiver operating characteristic curve analysis evaluating the usefulness of time progression as a marker for an eGFR rate < 60 mL/min/1.73 m^2^, the area under the curve (AUC) was 0.850 (95% CI 0.763-0.937, *p* < 0.001) (Figure 2). A time of evolution ≥ 15.5 years had a sensitivity of 85.7% and a specificity of 71.9% for a diagnosis of CKD.

Table 3 shows the differences between patients with equal to or more than 15.5 years of evolution versus those with a shorter duration of disease. Patients with a duration of disease ≥ 15.5 years were older (*p* = 0.002) and had lower serum creatinine (*p* = 0.006) and ΔeGFR (*p* = 0.009). On the other hand, patients with a longer evolution time had lower levels of albumin-adjusted serum calcium (*p* = 0.014), serum phosphorus (*p* = 0.024), PTH (*p* = 0.008), and a lower eGFR (*p* < 0.001). Doses of calcium carbonate were lower (*p* = 0.037) in patients with a longer duration of disease. Also, the percentage of patients with nephrocalcinosis (*p* < 0.049) and arrhythmia (*p* = 0.022) was higher in patients with ≥15.5 years of evolution.

## 4. Discussion

Our study showed that the eGFR was significantly associated with the duration of hypoparathyroidism. A disease duration of 15.5 years or more was associated with a significantly increased risk of decline in renal function. In addition, in patients with hypoparathyroidism, a history of hypertension, type 2 diabetes, and dyslipidemia was more prevalent.

Fourteen percent of patients in our cohort had an eGFR below < 60 mL/min/1.73 m^2^. Our data were consistent with findings from a Norwegian study [17], where 18% of the patients had an eGFR below 60 mL/min/1.73 m^2^. Other studies reported similar percentages of patients with CKD [18,19,20]. However, Mitchell et al. [21] found that the prevalence of CKD was 41%. In total, 44% of our patients presented hypercalciuria, 10% urolithiasis, and 6% nephrocalcinosis. In adult studies, rates for urolithiasis were reported using diagnosis codes (1–2%), ultrasound (8–30%), and self-reporting (35.5%). Meola et al. described that 30% of their patients had urolithiasis detected by renal ultrasound and that most were asymptomatic. Nephrocalcinosis rates varied from 0% to 38% [19].

We found an association of the eGFR with age and the duration of disease in our patients. Similar findings were found by Underbjerb et al., which may be related to an interaction between the age of the individual and the duration of disease, with a higher risk in elderly patients [22]. Also, Mitchel et al. [21] found that the eGFR was associated with age, duration of disease, and proportion of time with relative hypercalcemia. In this study, no association was found between eGFR levels and either 24 h urine calcium values or the presence of renal calcification [21]; we did not find a relationship with calciuria either. This could be due to thiazide treatment, as 29% of our patients underwent thiazide treatment. In previous studies that reported thiazide data, the percentages of patients who were prescribed this medication were 2% [20] and 20% [22].

We observed that the eGFR decreased in the presence of urolithiasis. Also, in patients with CKD, the incidence of urolithiasis and nephrocalcinosis was higher than in patients without CKD. Finally, the percentage of patients with nephrocalcinosis was higher in patients with a longer duration of disease. Swartling et al. found that the risk of developing de novo CKD and urolithiasis was higher in patients with chronic hypoparathyroidism compared to subjects without chronic hypoparathyroidism [23]. There was no difference in the risk of CKD between post-surgical and non-surgical etiologies. Additionally, they found that patients with chronic hypoparathyroidism had a higher incidence rate of hospitalization due to any cause, including CKD and urolithiasis [23].

During follow-up, a significant decrease in eGFR was observed over time, and there was a significant change in the diagnosis of CKD over time, with 14 new cases of CKD. Gosmanova et al. found that, compared to individuals without chronic hypoparathyroidism, subjects with chronic hypoparathyroidism also had a higher risk of significant deterioration in kidney function, progression of CKD, and development of end-stage renal disease [4]. A recent study conducted on a nationwide cohort in Spain showed that the deterioration of renal function over time was significantly greater in patients with chronic hypoparathyroidism following thyroidectomy compared to patients without hypoparathyroidism [24]. A similar finding was reported by Luk et al., who found that more patients with permanent hypoparathyroidism, with a median follow-up of 11.6 years, had a sustained 50% decrease in the eGFR from baseline compared to those without the disease [25]. We also found that a change in the eGFR is associated with the duration of disease, and the risk of CKD increases by 11% for each year of evolution of hypoparathyroidism.

The eGFR was found to decrease in the presence of hypertension, type 2 diabetes, and dyslipidemia. Patients with CKD had higher rates of hypertension, type 2 diabetes, dyslipidemia, coronary heart disease, and arrhythmia. Finally, the percentage of patients with arrhythmia was higher in patients with a longer disease duration. Fuss et al. found a higher prevalence of QTc interval prolongation and an increased incidence of hypertension [14]. Additionally, two studies reported the prevalence of hypertension and diabetes, with ranges from 3.5 to 18% and 1% to 8.4%, respectively [19,26]. The authors did not infer any relationship with renal outcomes based on the limited data. Studies conducted on small cohorts of patients with hypoparathyroidism demonstrated that hypocalcemia was associated with poor cardiovascular outcomes, including cardiomyopathy, congestive heart failure, and arrhythmia [23,27,28,29,30]. A cohort study on 8097 patients with chronic hypoparathyroidism compared to 40,485 patients without hypoparathyroidism found a significantly higher risk of incident cardiovascular conditions in patients with hypoparathyroidism [26]. Furthermore, the results of prospective clinical studies support this hypothesis regarding the relationship between calcium supplementation and CVD, including vascular calcification, stroke, coronary heart disease, and cardiovascular mortality [30,31,32]. Fibroblast growth factor 23 (FGF23) could be another potential causal factor of the increased CVD in patients with chronic hypoparathyroidism, although this has not been well studied in this population. FGF23 had been implicated in CVD in patients with CKD [33,34]. Emerging concern over potential CVD among patients with chronic hypoparathyroidism should be further investigated. Our data suggest that renal function in patients with chronic hypoparathyroidism is related to cardiovascular risk factors, such as hypertension, type 2 diabetes, and dyslipidemia, especially in older patients and patients with a longer duration of disease.

The duration of disease was associated with CKD. In the ROC curve analysis evaluating the usefulness of the time of progression as a marker for CKD, the AUC was significant. A time of evolution ≥ 15.5 years had a sensitivity of 85.7% and a specificity of 71.9% for a diagnosis of CKD. Previous studies had a mean duration of disease of 9 to 15.9 years, and the relationship between the duration of disease and renal function was studied. Mitchell et al., in their study on 120 patients, found that eGFR levels remained significantly associated with the duration of disease (*p* = 0.032) [21]. In a case-controlled retrospective study on 431 patients, an increased risk of any renal disease was associated with a longer duration of disease, which was a predictor of any incidence of renal disease (>12.7 vs. <12.7 years; *p* < 0.01) [19].

Conventional long-term treatment for chronic hypoparathyroidism corrects hypocalcemia by increasing intestinal calcium absorption, but does not affect renal calcium reabsorption or urinary phosphate excretion [1]. Therefore, it has been hypothesized that both the use of conventional treatment with active vitamin D and calcium supplements and the absence of PTH’s stimulating effects on calcium reabsorption in the renal tubules increase the risk of developing renal complications in patients with chronic hypoparathyroidism [4]. Treatments that replicate the physiologic effects of PTH via palopegteriparatide, reducing the need for conventional therapy, appeared to prevent a decline in renal function in this population [8]. Additionally, patients treated with PTH analogues had a lower risk of cardiovascular disease compared to patients not receiving PTH analogue therapy [15]. Emerging concern over potential CVD among patients with chronic hypoparathyroidism should be further investigated. The latest clinical guidelines for the evaluation and management of hypoparathyroidism suggest the use of PTH replacement therapy in patients who are not adequately controlled with conventional therapy. Also, it is recommended for individuals with poor compliance, malabsorption, intolerance to large doses of calcium and active vitamin D, or renal insufficiency [5].

Our study has some limitations. First, the observational design does not allow for the establishment of a cause–effect relationship. Moreover, our study population included only Spanish Caucasian individuals from a specific area. In addition, we did not include a control group to compare our findings. However, our work has several strengths. Our retrospective longitudinal study allowed us to conduct a comprehensive evaluation of clinical and biochemical parameters, integrating all variables that could influence cardiovascular risk in a large sample of patients with chronic hypoparathyroidism. In addition, we performed rigorous statistical analyses to obtain reliable results. Furthermore, we postulated the diagnostic usefulness of the duration of chronic hypoparathyroidism in the diagnosis of CKD.

## 5. Conclusions

The duration of disease appears to be the main predictor of the presence of renal dysfunction in patients with chronic hypoparathyroidism. In addition, the coexistence of CVD factors could result in greater renal damage.

## Figures and Tables

**Table 1:** Characteristics of patient cohort.

	Population Study	Range
Age (years)	57 (14)	29–86
Duration of disease (years)	15 (11)	1–54
Serum calcium (mg/dL)	8.6 (0.7)	6.3–10.0
Albumin-adjusted serum calcium levels (mg/dL)	8.3 (0.7)	6.2–9.9
Serum phosphorus (mg/dL)	4.3 (0.3)	2.5–6.5
Serum magnesium (mg/dL)	1.8 (0.2)	1.1–2.3
Calcium phosphate product (mg^2^/dL^2^)	35.7 (5.9)	19.8–61.7
PTH levels (mg/dL)	11.5 (11.6)	1.0–49.0
25 OH vitamin D (mg/dL)	32.3 (12)	13.0–63.0
Creatinine (mg/dL)	0.86 (0.27)	0.56–1.81
eGFR (mL/min/1.73 m^2^)	78.2 (17.2)	28.1–91.8
ΔeGFR (mL/min/1.73 m^2^)	−10.36 (16.63)	−61.88–11.00
ΔeGFR/yr (ΔeGFR/year)	−0.64 (1.04)	−4.13–2.75
Calciuria (mg/24 h)	243.9 (137.9)	5.4–635.0
Hypercalciuria	44%	NA
Urolithiasis	10%	NA
Nephrocalcinosis	6%	NA
eGFR < 60 mL/min/1.73 m^2^	14%	NA
Hypertension	38%	NA
Type 2 diabetes	18%	NA
Dyslipidemia	44%	NA
Coronary heart disease	3%	NA
Cerebrovascular disease	3%	NA
Peripheral arterial disease	2%	NA
Arrhythmia	3%	NA

eGFR: Estimated glomerular filtration rate; ΔeGFR: change in eGFR (ΔeGFR = eGFR at last visit—eGFR at diagnosis); ΔeGFR/yr: change in eGFR corrected for duration of follow-up (ΔeGFR/yr = ΔeGFR/time in years). The data for continuous variables are presented as the mean (SD). The data for categorical variables are presented as percentages. NA, no applicable.

**Table 2:** Characteristics and differences between patients with eGFR ≥ 60 mL/min/1.72 m^2^ and patients with eGFR < 60 mL/min/1.72 m^2^.

	eGFR ≥ 60 mL/min/1.72 m^2^ n: 86	eGFR < 60 mL/min/1.72 m^2^ n: 14	*p* Value
Etiology post-surgery (%)	89.5	87.5	0.672
Age (years)	55 (13)	75 (7)	**<0.001**
Gender (% females)	86	85	0.468
Duration of disease (years)	13 (10)	27 (11)	**<0.001**
Serum calcium (mg/dL)	8.5 (0.7)	9.1 (0.7)	**<0.001**
Albumin-adjusted serum calcium levels (mg/dL)	8.2 (0.7)	8.9 (0.7)	**<0.001**
Serum phosphorus (mg/dL)	4.3 (0.7)	3.8 (0.5)	**0.002**
Serum magnesium (mg/dL)	1.8 (0.2)	1.8 (0.3)	0.441
Calcium phosphate product (mg^2^/dL^2^)	36.0 (6.0)	33.9 (4.9)	0.662
PTH levels (mg/dL)	11.9 (11.5)	9.2 (11.3)	0.383
25 OH vitamin D (mg/dL)	32.8 (12.0)	28.9 (11.0)	0.975
Creatinine (mg/dL)	0.78 (0.14)	1.38 (0.27)	**<0.001**
eGFR (mL/min/1.73 m^2^)	84.2 (8.1)	40.9 (9.2)	**<0.001**
ΔeGFR (mL/min/1.73 m^2^)	−4.6 (8.4)	−44.0 (12.0)	**<0.001**
ΔeGFR/yr (ΔeGFR/year)	−0.43 (0.89)	−1.90 (0.92)	**<0.001**
Calciuria (mg/24 h)	248.1(135.6)	219.4 (154.4)	0.864
Calcium carbonate doses (mg/24 h)	2311 (1592)	1713 (785)	0.213
Magnesium treatment (%) Doses (mg/24 h)	20.9 187 (120)	28.6 87 (74)	0.522 0.423
Calcitriol treatment doses (mcg/24 h)	0.627 (0.494)	0.404 (0.127)	0.101
Colecalciferol treatment (%) Doses (UI/24 h)	57.0 1944 (3474)	42.9 893 (454)	0.325 0.432
Calcifediol treatment (%) Doses (mcg/24 h)	10.5 0.296 (0.088)	7.7 0.266	0.757 -
Tiazide treatment (%) Doses (mg/24 h)	27.9 22.4 (13.8)	35.7 27.5 (13.7)	0.342 0.684
Hypercalciuria (%)	45.0	37.7	0.487
Urolithiasis (%)	7.0	28.6	**0.003**
Nephrocalcinosis (%)	4.7	14.3	**0.008**
Hypertension (%)	32.6	71.4	**<0.005**
Type 2 diabetes (%)	12.8	50	**<0.001**
Dyslipidemia (%)	36.0	92.2	**<0.001**
Coronary disease (%)	1.2	14.3	**0.008**
Cerebrovascular disease (%)	2.3	7.1	0.327
Peripheral arterial disease (%)	1.2	7.1	0.138
Arrhythmia (%)	0	21.4	**<0.001**

PTH: parathormone; eGFR: estimated glomerular filtration rate; ΔeGFR: change in eGFR (ΔeGFR = eGFR at last visit—eGFR at diagnosis); ΔeGFR/yr: change in eGFR corrected for duration of follow-up (ΔeGFR/yr = ΔeGFR/time in years). The data for continuous variables are presented as the mean (SD). The data for categorical variables are presented as percentages. Unpaired Student’s *t*-test and U Mann–Whitney test were used for comparisons of continuous variables between groups. The χ^2^ test was used for the comparison of categorical variables between groups.

**Table 3:** Differences between those patients with more than 15.5 years of evolution versus those with a shorter evolution time.

	<15.5 Years of Evolution n: 63	≥15.5 Years Evolution n: 37	*p* Value
Etiology post-surgery (%)	94	81	**0.052**
Age (years)	54 (13)	63 (14)	**0.002**
Gender (% females)	86	84	0.794
Duration of disease (years)	8.5 (4.1)	26.7 (9.6)	**<0.001**
Serum calcium (mg/dL)	8.8 (0.6)	8.5 (0.7)	**0.031**
Albumin-adjusted serum calcium levels (mg/dL)	8.5 (0.7)	8.2 (0.7)	**0.014**
Serum phosphorus (mg/dL)	4.4 (0.7)	4.1 (0.6)	**0.024**
Serum magnesium (mg/dL)	1.8 (0.2)	1.8 (0.2)	0.125
Calcium phosphate product (mg2/dL2)	36 (6)	35.2 (5.8)	0.892
PTH levels (mg/dL)	13.5 (12.5)	8.3 (8.6)	**0.008**
25 OH vitamin D (mg/dL)	32.7 (11.1)	31.7 (13.3)	0.111
Creatinine (mg/dL)	0.8 (0.22)	0.96 (0.31)	**0.006**
eGFR (mL/min/1.73)	82.1 (12.8)	71.4 (21.4)	**<0.001**
ΔeGFR (mL/min/1.73 m^2^)	−6.5 (12.3)	−17.1 (20.8)	**0.009**
ΔeGFR/yr (ΔeGFR/year)	−0.63 (1.15)	−0.66 (0.8)	0.365
Calciuria (mg/24 h)	244.3 (146.7)	243 (124)	0.217
Calcium carbonate doses (mg/24 h)	2408 (1739)	1915 (960)	**0.037**
Magnesium treatment (%) Doses (mg/24 h)	22.2 208 (127)	21.6 103 (68)	0.922 **0.022**
Calcitriol treatment doses (mcg/24 h)	0.644 (0.57)	0.514 (0.191)	0.13
Colecalciferol treatment (%) Doses (UI/24 h)	54 1405 (836)	56.8 2514 (5231)	0.787 0.59
Calcifediol treatment (%) Doses (mg/24 h)	14.3 0.296 (0.088)	2.8 0.266	0.068 -
Tiazide treatment (%) Doses (mg/24 h)	25.4 24.2 (16.1)	35.1 22.1 (10.4)	0.3 0.61
Hypercalciuria (%)	42.9	45.9	0.875
Urolithiasis (%)	7.9	13.5	0.085
Nephrocalcinosis (%)	3.2	10.8	**0.049**
Hypertension (%)	31.7	48.6	0.093
Type 2 diabetes (%)	12.7	27	0.072
Dyslipidemia (%)	38.1	54.1	0.121
Coronary disease (%)	1.6	5.4	0.28
Cerebrovascular disease (%)	1.6	5.4	0.28
Peripheral arterial disease (%)	1.6	2.7	0.7
Arrhythmia (%)	0	8.1	**0.022**

PTH: parathormone; eGFR: estimated glomerular filtration rate; ΔeGFR: change in eGFR (ΔeGFR = eGFR at last visit–eGFR at diagnosis); ΔeGFR/yr: change in eGFR corrected for duration of follow-up (ΔeGFR/yr = ΔeGFR/time in years). The data for continuous variables are presented as the mean (SD). The data for categorical variables are presented as percentages. Unpaired Student’s *t*-test and U Mann–Whitney test were used for comparisons of continuous variables between groups. The χ^2^ test was used for the comparison of categorical variables between groups.

## Data Availability

The original contributions presented in the study are included in the article, further inquiries can be directed to the corresponding authors.
